# Cell wall structures leading to cultivar differences in softening rates develop early during apple (Malus x domestica) fruit growth

**DOI:** 10.1186/1471-2229-13-183

**Published:** 2013-11-19

**Authors:** Jovyn KT Ng, Roswitha Schröder, Paul W Sutherland, Ian C Hallett, Miriam I Hall, Roneel Prakash, Bronwen G Smith, Laurence D Melton, Jason W Johnston

**Affiliations:** 1 Food Science, School of Chemical Sciences, The University of Auckland, Private Bag 92019, Auckland, New Zealand; 2 The New Zealand Institute for Plant & Food Research Limited, Mount Albert Research Centre, Private Bag 92169, Auckland 1142, New Zealand; 3 Current address: The New Zealand Institute for Plant & Food Research Limited, Food Industry Science Centre, Private Bag 11600, Palmerston North 4442, New Zealand; 4 The New Zealand Institute for Plant & Food Research Limited, Hawkes Bay Research Centre, Havelock North 4130, New Zealand

**Keywords:** Apple, Cell adhesion, Cell wall, Fruit firmness, Immunofluorescence labelling, Microstructure, Pectin

## Abstract

**Background:**

There is a paucity of information regarding development of fruit tissue microstructure and changes in the cell walls during fruit growth, and how these developmental processes differ between cultivars with contrasting softening behaviour. In this study we compare two apple cultivars that show different softening rates during fruit development and ripening. We investigate whether these different softening behaviours manifest themselves late during ethylene-induced softening in the ripening phase, or early during fruit expansion and maturation.

**Results:**

‘Scifresh’ (slow softening) and ‘Royal Gala’ (rapid softening) apples show differences in cortical microstructure and cell adhesion as early as the cell expansion phase. ‘Scifresh’ apples showed reduced loss of firmness and greater dry matter accumulation compared with ‘Royal Gala’ during early fruit development, suggesting differences in resource allocation that influence tissue structural properties. Tricellular junctions in ‘Scifresh’ were rich in highly-esterified pectin, contributing to stronger cell adhesion and an increased resistance to the development of large airspaces during cell expansion. Consequently, mature fruit of ‘Scifresh’ showed larger, more angular shaped cells than ‘Royal Gala’, with less airspaces and denser tissue. Stronger cell adhesion in ripe ‘Scifresh’ resulted in tissue fracture by cell rupture rather than by cell-to-cell-separation as seen in ‘Royal Gala’. CDTA-soluble pectin differed in both cultivars during development, implicating its involvement in cell adhesion. Low pectin methylesterase activity during early stages of fruit development coupled with the lack of immuno-detectable PG was associated with increased cell adhesion in ‘Scifresh’.

**Conclusions:**

Our results indicate that cell wall structures leading to differences in softening rates of apple fruit develop early during fruit growth and well before the induction of the ripening process.

## Background

Apple cultivars exhibit variable rates of softening during ripening and can vary in firmness once mature [1]. Our aim was to determine when these differences in softening behaviour in apple fruit manifest themselves; in the early stages of fruit development, after cell division ceases and cell expansion starts, giving rise to the development of the complex three-dimensional cortical tissue; or later in development, when the fruit approaches full size and begins to initiate ripening. In this study, we explore the role of cortical microstructure in the softening behaviour of apples throughout development and ripening.

Apples undergo two distinct phases during growth: a phase of intensive cell division which lasts typically 3-5 weeks after full bloom, followed by a phase of cell expansion when cell division ceases [2]. As the fruit grows and increases in size, mass is gained mainly through water uptake and increase in parenchyma cell volume [3]. Firmness declines during fruit expansion, which coincides with reduced density of cell packing and increased cell volume and air spaces [4]. Thus, the cell wall not only has to maintain structural integrity during growth, but also allow expansion of cell size and associated extracellular air spaces. There is currently a paucity of information regarding development of tissue microstructure and changes in the cell walls during fruit growth, let alone how these developmental processes differ between cultivars with contrasting softening behaviour. Instead, considerable attention has been paid to changes in cell wall chemistry and microstructure [5,6] during ripening, however this has only limited applicability for understanding when important microstructural features develop during growth.

Assessment of tissue microstructure is complex, as it is influenced by many different cellular components, and multiple, complementary approaches are needed to develop a robust view of how cultivars differ. Studies using apple mapping populations to investigate the relationship between textural properties and cell size and shape have produced inconsistent results, with one study failing to detect quantitative trait loci (QTL) for cell size despite detecting QTLs for textural properties [7]. In contrast, a second study showed a significant correlation between cell size and textural properties [8]. Further studies using ripe commercial apple cultivars have reinforced the association between cell size and texture, where increased sensory juiciness was associated with larger cell sizes and more densely packed tissue [9].

Another important aspect of tissue microstructure is the interconnections between adjacent cells, and how they contribute to a three-dimensional structure. Cell-to-cell adhesion is important, as it affects the fracture path across tissues and contributes to the mode of tissue failure. The examination of fracture surfaces for apples, and a broader survey across different types of fruit have shown two main types of tissue failure: 1) cell rupture that results in the release of cellular contents; and 2) cell-to-cell separation where adjacent cells separate without cell rupture [10-12]. It has also been shown that cell rupture can be classified as equatorial, or as a top or bottom fracture which may affect the rate at which cell contents are released [9]. These results reinforce the need to understand the chemistry of the cell wall in terms of dissolution of the middle lamella and the separation of adjacent cells, while taking into consideration the spatial distribution of different types of pectin in the cell junction zones during softening.

Homogalacturonan (HG) pectin is believed to play a major role in intercellular adhesion, as it is commonly found in the middle lamella region of the cell wall where two adjacent cells adjoin [13]. Here, stretches of un-esterified galacturonic acid residues of HG are thought to provide the main bonding between adjacent cells through calcium cross-links [14]. Enzymes that are likely to play a role in modulating cell adhesion properties are pectin methylesterase (PME) and endo-polygalacturonase (PG), where PG is thought to depolymerise HG within stretches of un-esterified galacturonic acid residues created by PME [15].

A number of studies have reported on the occurrence and activity of PG in apples [16-19] but the low abundance of PG protein and in most cases undetectable enzymatic activity has shifted research towards a transgenic approach [20-22]. Expression of the ripening-related *MdPG1* gene in ‘Royal Gala’ is induced during cold storage [23], and its down-regulation in ‘Royal Gala’ increased cell adhesion and reduced softening [21], whereas over-expression of the same gene led to increased intercellular separation in ‘Royal Gala’ leaves [20], demonstrating a role for PG in the loss of intercellular adhesion. PME protein is found in most plant tissues and exists in multiple isoforms. In apple, PME activity has been found to increase during growth and decrease during ripening-related softening [24], but its role is less clear than that of PG. Apart from pectic-related changes, xyloglucan and enzymes such as xyloglucan endotransglucosylase/hydrolase (XTH) also play important roles in the development of apple fruit texture and softening [8,25]. Recent work in apple has shown an increase in XTH gene expression induced by ethylene, thereby emphasizing the role of XTH in xyloglucan modification during apple fruit softening [26].

In this paper, we determine the microstructural properties throughout fruit development and softening of ‘Scifresh’ (commercially marketed as Jazz™), an apple cultivar that loses firmness slowly during ripening despite high ethylene production, and compare it with ‘Royal Gala’, a parent of ‘Scifresh’, that softens more rapidly during ripening but has a similarly high ethylene production. The advantage of this approach is that the ripening phenotype is not confounded by differences in ethylene production, enabling a more robust assessment of the relative contribution of structural features towards softening. By using a combination of different techniques, we investigate differences between the cultivars in cell size and cell packing, fracture pattern, tensile strength and cell-to-cell adhesion. Immunolocalisation, cell wall fractionation and size exclusion chromatography are used to examine differences in pectin between adjacent cells and in zones where extracellular air spaces develop during growth and ripening. The involvement of pectin-modifying enzymes pectin methylesterase (PME) and polygalacturonase (PG) are also investigated.

## Results

### ‘Scifresh’ And ‘royal Gala’ differ in a range of physiological parameters during growth and ripening

**Figure 1:**
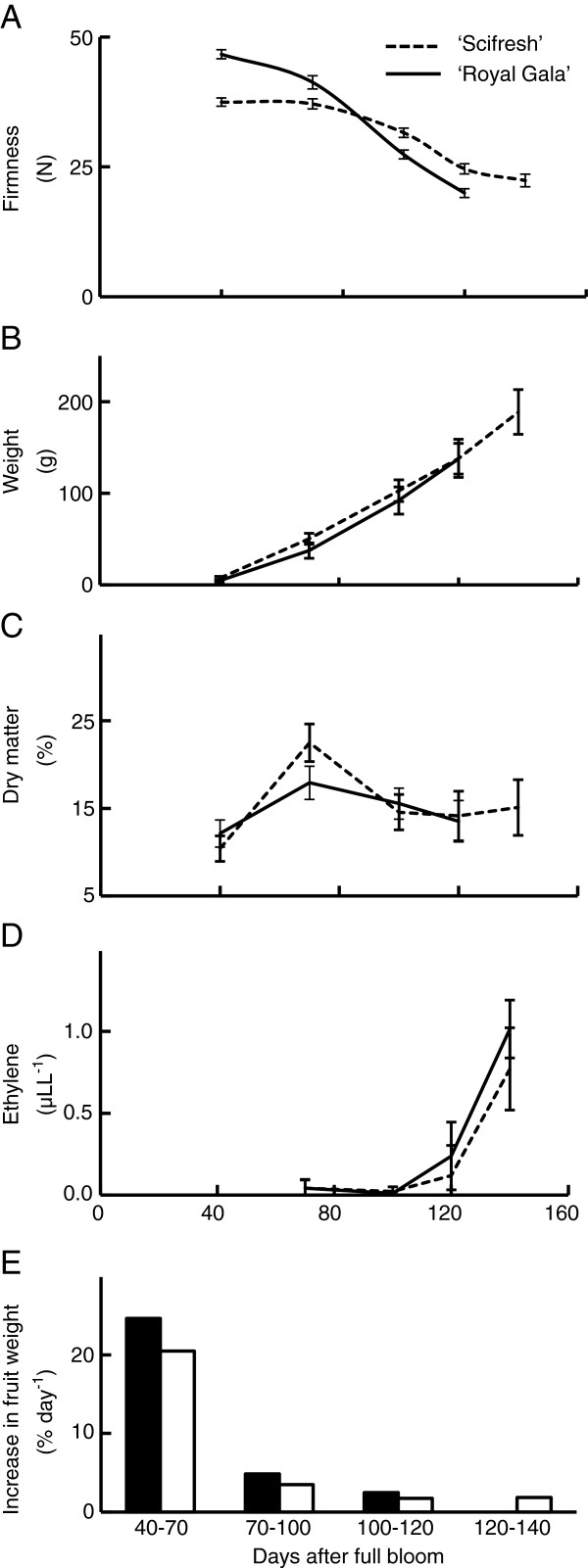
**Physiological parameters during growth and maturation of ‘Royal Gala’ and ‘Scifresh’.** Flesh firmness **(A)**, fruit weight **(B)**, dry matter concentration **(C)**, internal ethylene production **(D)**, and percent increase in fruit weight **(E)** during development of ‘Royal Gala’ and ‘Scifresh’ apples (*n* = 20 ± SE). Probe size for measuring firmness was 5 mm. The percentage increase in fruit weight **(E)** was calculated as the change in mean fruit weight relative to the weight at the start of each period per day.

**Figure 2:**
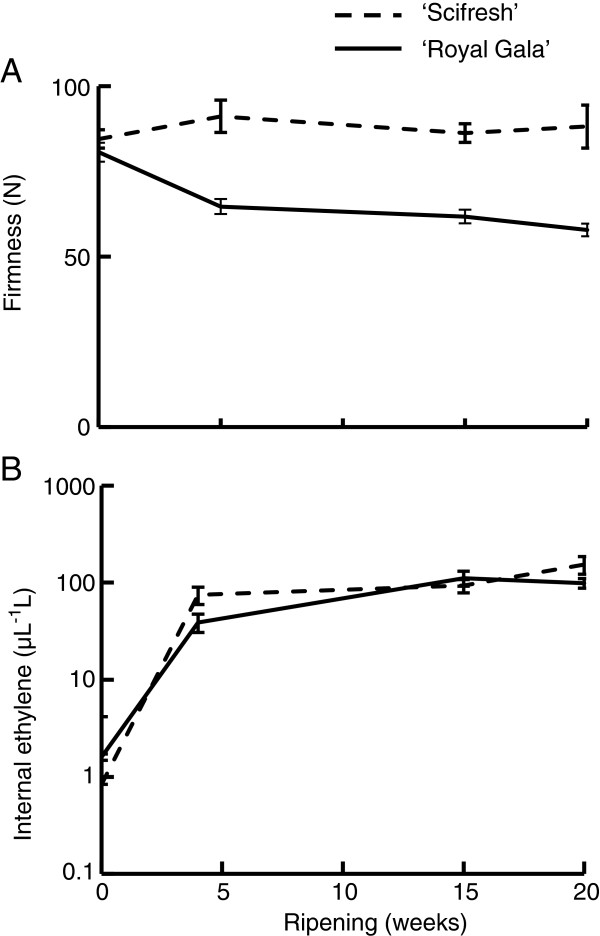
**Firmness loss and ethylene production of ‘Royal Gala’ and ‘Scifresh’ fruit during ripening.** Flesh firmness **(A)** and internal ethylene production **(B)** during ripening at 0.5°C (*n* = 20 ± SE). Note: probe size for measuring firmness was 11 mm.

Firmness of both apple cultivars declined during fruit growth, but differed in initial firmness and the subsequent rate of decline. ‘Royal Gala’ fruitlets were firmer than ‘Scifresh’ at 40 DAFB, but then softened faster to a lower firmness once mature (Figure 1A). ‘Scifresh’ apples had a lag phase with minimal loss of firmness between 40 and 70 DAFB. Both cultivars had a similar increase of fruit weight (Figure 1B), which coincided with the decline in firmness. However, firmness decline was not exclusively due to fruit growth, as the firmness lag phase for ‘Scifresh’ was not accompanied by slower growth. Dry weight accumulation was similar for both cultivars, with the only difference occurring 70 DAFB when ‘Scifresh’ accumulated more dry matter than ‘Royal Gala’ (Figure 1C). For ‘Scifresh’, this peak in dry matter accumulation coincided with the end of the lag phase for loss of firmness (Figure 1A) and the rapid growth phase (Figure 1E), suggesting cultivar differences in dry weight assimilation and partitioning into structural features during fruit growth. Fruit internal ethylene concentrations were comparable between the two cultivars during fruit growth and maturation (Figure 1D), with the climacteric rise in ethylene occurring between 100 and 120 DAFB. The two cultivars also produced similar concentrations of ethylene during ripening, but had substantial differences in the rate of softening (Figure 2). ‘Royal Gala’ declined in firmness by ca. 35% during ripening, while ‘Scifresh’ effectively did not change in firmness over the same period (Figure 2A).

### ‘Scifresh’ cortex tissue has larger cells and is more dense than ‘Royal Gala’

**Figure 3:**
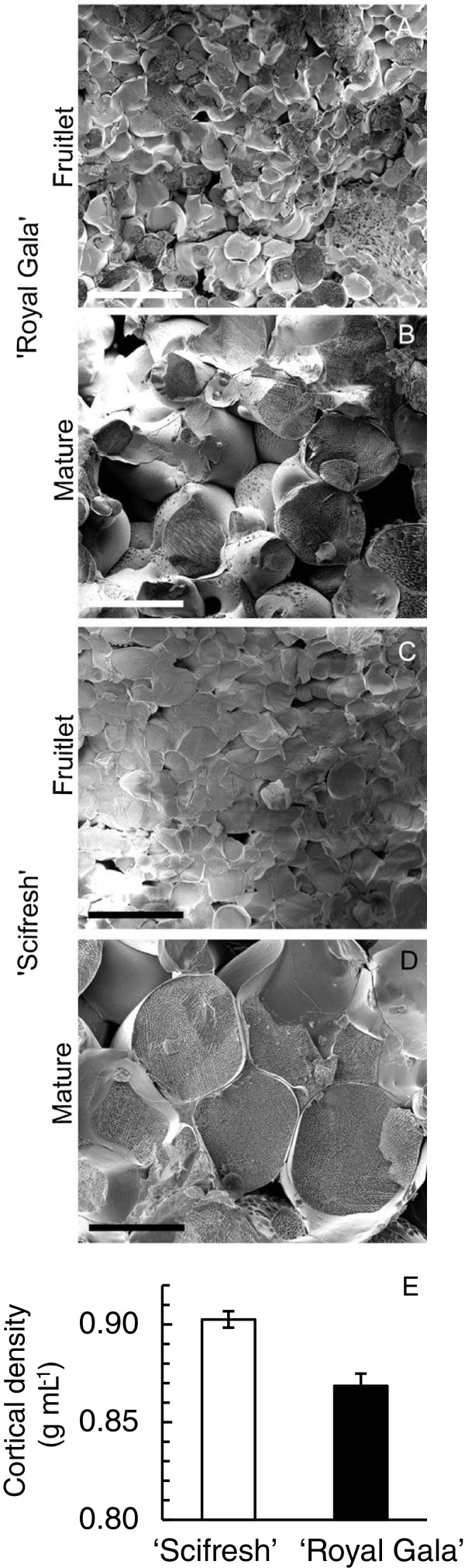
**Size and density of cortical cells of ‘Royal Gala’ and ‘Scifresh’ at the fruitlet and mature stage.** Cryo-scanning electron micrographs of fruitlet **(A, C)** and mature fruit **(B, D)** of ‘Royal Gala’ **(A, B)** and ‘Scifresh’ **(C, D)**, and cortical density of cells at the mature fruit stage **(E)**. Bars = 200 μm for all micrographs. Apples were matched for size at each stage of development. Values in **(E)** are the mean of 15 measurements ± SE.

Cryo-scanning electron micrographs of cortex tissue showed that both cultivars had similar cell size at the fruitlet stage (Figure 3A, C), but cells were larger in ‘Scifresh’ than in ‘Royal Gala’ at the mature stage (Figure 3B, D) for fruit of equivalent size. Mature ‘Scifresh’ fruit had an average cell diameter of 166 ± 13.8 μm (*n* = 15), while mature ‘Royal Gala’ fruit had an average cell diameter of 107 ± 11.7 μm (*n* = 15). These findings were in agreement with [27] who found, using a different method, that ‘Scifresh’ cells were 49% larger by area than ‘Royal Gala’. Our results show that the cells in ‘Scifresh’ expanded at a greater rate during fruit growth than in ‘Royal Gala’, yet both cultivars result in a similar fruit size. In both cultivars, development of larger intercellular air spaces was observed between the fruitlet and mature stage (Figure 3). An estimate of air space volume showed that ‘Scifresh’ had a higher cortical tissue density than ‘Royal Gala’, once fruit were mature (Figure 3E).

### Fracture surfaces of ripe tissue shows more cell rupture and greater cell adhesion in ‘Scifresh’

**Figure 4:**
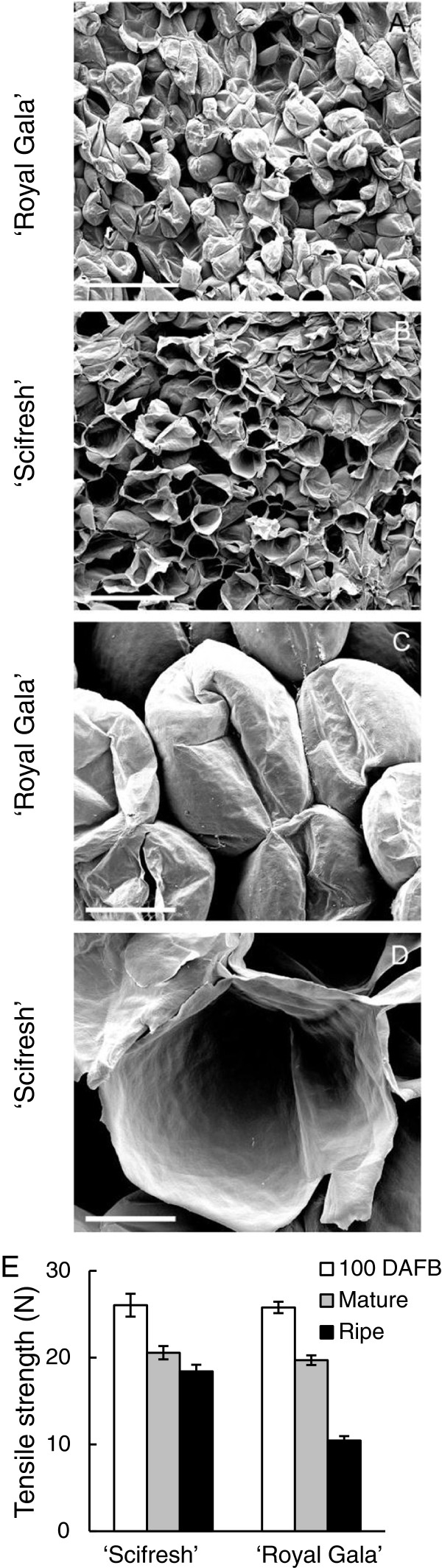
**Fracture pattern and tensile strength of cortical tissue of ‘Royal Gala’ and ‘Scifresh’ fruit during ripening.** Scanning electron micrographs of ripe ‘Royal Gala’ **(A, C)** and ‘Scifresh’ fruit **(B, D)** (20 weeks at 0.5°C), showing a different fracture pattern between cells with the appearance of more intact cells in ‘Royal Gala’ and more broken open cells in ‘Scifresh’. **(E)** Tensile tests to quantify the force required to pull cortex tissue apart (expanding fruit 100 DAFB, mature and ripe fruit). Bars **A**, **B** = 500 μm; bars **C**, **D** = 100 μm. Values in **(E)** are the mean of 15 measurements ± SE.

Using conventional scanning electron microscopy, distinct differences in the fracture pattern were observed in ripe tissue (Figure 4A–D). ‘Royal Gala’ tissue primarily fractured between cells resulting in minimal cell rupture (Figure 4A, C), while fracturing of ‘Scifresh’ tissue occurred more by cell rupture with minimal evidence of cell separation between adjacent cells (Figure 4B, D). Tensile tests were used to quantify the force required to pull cortex tissue apart. At earlier stages of development (100 DAFB and mature) the tensile properties of the two cultivars were similar, with both having loss of tensile strength during the final stages of fruit growth (100 DAFB to maturity). Once ripe, ‘Royal Gala’ required 50% less force than ‘Scifresh’ to fracture the tissue (Figure 4E). These tensile properties support the fracture surface images (Figure 4A–D) by showing that cell separation is associated with weak adhesion forces between adjacent cells in ‘Royal Gala’.

### Cellular junctions are filled with highly esterified pectin in ‘Scifresh’ throughout development, but not in ‘Royal Gala’

**Figure 5:**
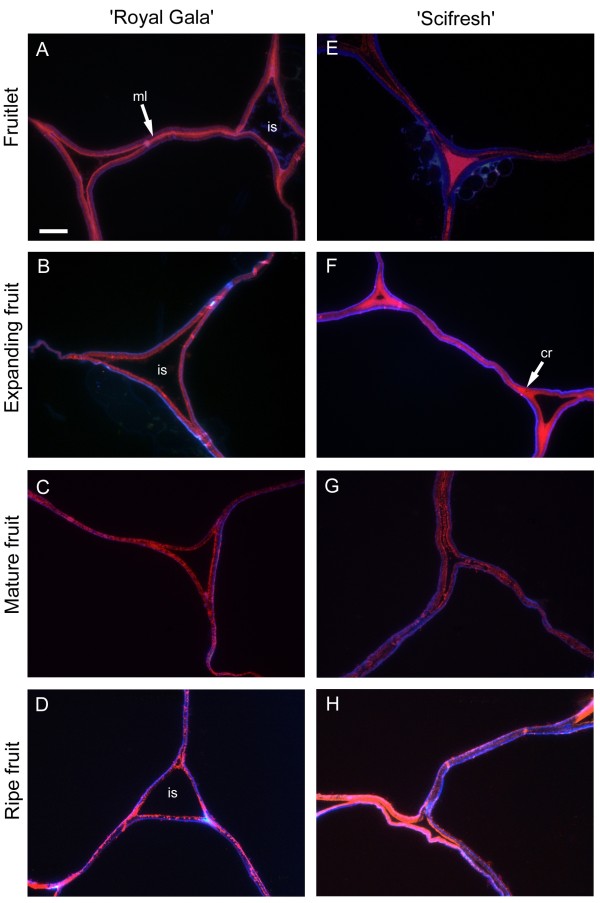
**Immunofluorescence labelling for lowly-esterified homogalacturonan during apple fruit development.** LM19 antibody labelling (pink) and anti-cellulose stain calcofluor (blue) in ‘Royal Gala’ **(A-D)** and ‘Scifresh’ **(E-H)** apple cortex tissue. Fruitlet: 40 DAFB; Expanding fruit: 70 DAFB; Mature fruit: 120 DAFB (RG) 140 DAFB (SF); Ripe fruit: 20 weeks at 0.5°C. Bar in **(A)** = 10 μm for all micrographs. cr, corner of tricellular junction; ml, middle lamella; is intercellular space.

The monoclonal antibody LM19, specific for non- or low methyl-esterified HG regions of pectin, labelled cell walls in tissue of both cultivars in two distinct patterns, in the middle lamella region and in the corners of tricellular junctions (Figure 5; pink labelling). The labelling patterns were similar in both cultivars and were most pronounced in fruitlet and expanding fruits, and weakest in mature and ripe fruit. In ‘Scifresh’, intercellular spaces seemed smaller than in ‘Royal Gala’ at all developmental stages, supporting density measurements (Figure 3E). The intercellular spaces, particularly its corners in ‘Scifresh’ at the fruitlet and expanding stage were filled with non- or low-esterified pectin, whereas ‘Royal Gala’ seemed to have less of this epitope filling the intercellular spaces (Figure 5). Co-staining of sections with calcofluor (Figure 5; blue labelling) showed cellulose staining around the inside of the cell walls towards the cell lumen in both cultivars. Additional file 1: Figure S1 shows the LM19 antibody-labelling in green fluorescence at lower magnification.

**Figure 6:**
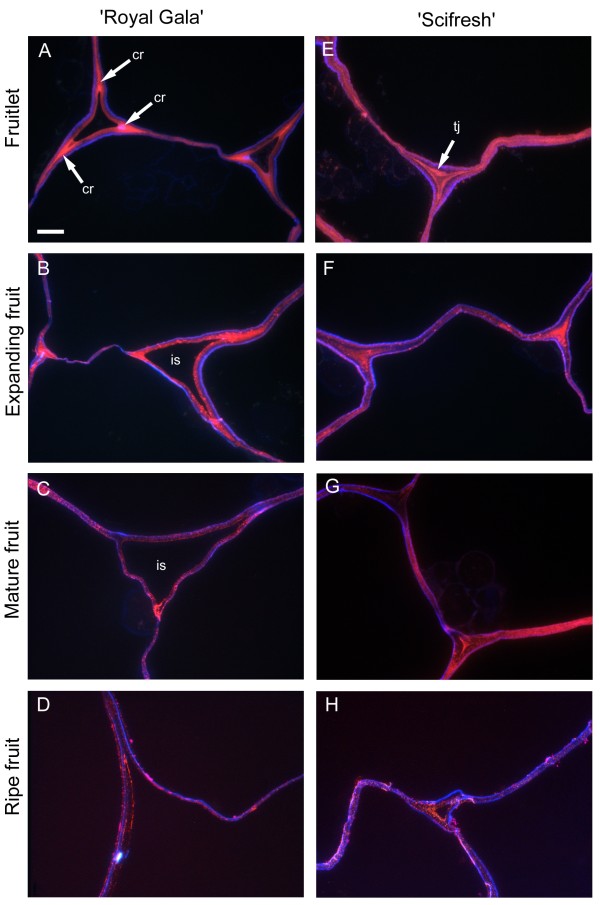
**Immunofluorescence labelling for highly-esterified homogalacturonan during apple fruit development.** LM20 antibody labelling (pink) and anti-cellulose stain calcofluor (blue) in ‘Royal Gala’ **(A-D)** and ‘Scifresh’ **(E-H)** apple cortex tissue. Fruitlet: 40 DAFB; Expanding fruit: 70 DAFB; Mature fruit: 120 DAFB (RG) 140 DAFB (SF); Ripe fruit: 20 weeks at 0.5°C. Bar in **(A)** = 10 μm for all micrographs. cr, corner of tricellular junction; tj, tricellular junction; is, intercellular space.

LM20, specific for more highly methyl-esterified HG regions, labelled ‘Royal Gala’ fruitlet and expanding fruit intensely in corners of tricellular junctions, whereas in the more tightly-packed cells of ‘Scifresh’, this epitope occurred more throughout junction zones and was not just restricted to the corners (Figure 6, pink labelling). In ‘Scifresh’, the middle lamella region also labelled more intensely, indicating more esterified HG in these areas compared to ‘Royal Gala’. In ‘Royal Gala’, intercellular spaces enlarged during fruit growth, whereas in ‘Scifresh’, they remained similar to the fruitlet stage. As with the LM19, the intercellular spaces of ‘Scifresh’ fruitlet and expanding fruit were filled with esterified pectin (Figure 6). Additionally, this intense labelling of esterified pectin in tricellular junctions remained up to maturation in ‘Scifresh’ fruit, whereas mature ‘Royal Gala’ fruit displayed weaker labelling. In ripe fruit of both cultivars, labelling became weaker, although still concentrated at the middle lamella region, but absent from the intercellular space which filled tricellular junctions. Co-staining of sections with calcofluor (Figure 6; blue labelling) showed cellulose staining around the inside of the cell walls towards the cell lumen in both cultivars. Additional file 2: Figure S2 and Additional file 3: Figure S3 show LM20 antibody-labelling in green fluorescence.

**Figure 7:**
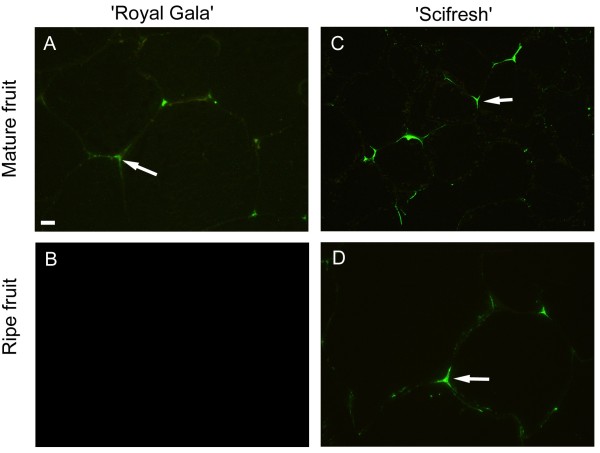
**Immunofluorescence labelling for calcium-associated HG regions with antibody 2F4 in mature and ripe apple fruit.** ‘Royal Gala’ **(A, B)** and ‘Scifresh’ **(C, D)** apple cortex tissue of mature fruit and ripe fruit. 2F4-labelling (green) was concentrated at tricellular junctions as indicated by the arrows. No labelling was detected in ripe ‘Royal Gala’ **(B)**. Bar in **(A)** = 10 μm for all micrographs.

HG regions with sufficiently low degree of methyl-esterification (<40%) and with at least nine consecutive non-esterified galacturonic acid residues can interact with divalent cations like calcium [28]. The monoclonal antibody 2F4 is specific to these calcium cross-linked HG epitopes. Labelling of cell walls with 2F4 was detected in mature fruit of both cultivars, but disappeared during softening in ‘Royal Gala’ and remained in ‘Scifresh’ (Figure 7). In both cultivars labelling was restricted to junction zones, particularly tricellular junctions. No labelling was found throughout the cell walls and very little was detected in the middle lamella, confirming that calcium cross-linked HG epitopes are restricted to the middle lamellae at cell corners and pit fields [29]. No labelling was detected in fruitlets of both cultivars (not shown), while expanding fruit were not examined.

### Cell wall yield and composition differed most between cultivars during early stages of fruit growth

The amount of cell wall material on a fresh weight basis was highest in fruitlet and decreased during growth, maturation and ripening in both cultivars (Table 1). Apart from the fruitlet stage, where the yield of cell wall material was considerably higher in ‘Royal Gala’ than in ‘Scifresh’, yields were comparable in both cultivars. The sum of the pectin-related sugars uronic acid, rhamnose, arabinose and galactose indicated a substantial amount of pectin of over 80% of the total amount of non-cellulosic sugars (Table 1).

**Table 1:** Yields and composition of cell wall material of fruitlet, expanding, mature and ripe ‘Royal Gala’ (RG) and ‘Scifresh’ (SF) apples

**Stage**	**Fruitlet**		**Expanding**		**Mature**		**Ripe**	
	**40 DAFB**		**70 DAFB**		**120/140 DAFB**		**20 weeks**	
**Cultivar**	**RG**	**SF**	**RG**	**SF**	**RG**	**SF**	**RG**	**SF**
Yield cell wall material	64.3 ± 5.3	51.2 ± 8.3	19.9 ± 2.2	20.4 ± 4.2	16.1 ± 3.6	17.1 ± 3.54	7.1 ± 2.9	9.4 ± 2.2
(mg g^-1^ FW ± SD)								
Sugars (mg g^-1^ FW ± SD)								
Rha	0.6 ± 0.05	0.4 ± 0.04	0.17 ± 0.01	0.13 ± 0.02	0.15 ± 0.02	0.12 ± 0.02	trace	trace
Fuc	0.2 ± 0.02	0.2 ± 0.01	trace	trace	trace	trace	trace	trace
Ara	5.1 ± 0.29	5.0 ± 0.3	1.5 ± 0.1	1.4 ± 0.2	1.2 ± 0.09	1.1 ± 0.05	0.3 ± 0.03	0.4 ± 0.02
Xyl	0.9 ± 0.1	1.1 ± 0.02	0.4 ± 0.07	0.4 ± 0.05	0.4 ± 0.04	0.5 ± 0.03	0.3 ± 0.02	0.3 ± 0.01
Man	0.5 ± 0.03	0.7 ± 0.05	0.2 ± 0.02	0.2 ± 0.02	0.1 ± 0.02	0.1 ± 0.01	trace	trace
Gal	5.5 ± 0.4	6.8 ± 0.5	2.3 ± 0.1	2.4 ± 0.4	1.1 ± 0.1	1.7 ± 0.06	0.3 ± 0.01	0.6 ± 0.08
Glc	0.7 ± 0.06	0.8 ± 0.1	0.3 ± 0.2	0.5 ± 0.02	0.3 ± 0.02	0.4 ± 0.13	0.14 ± 0.01	0.17 ± 0.02
UA	13.4 ± 2.1	7.0 ± 2.2	4.0 ± 0.3	3.6 ± 0.4	3.3 ± 0.8	3.1 ± 0.22	1.5 ± 0.1	2.2 ± 0.2
Total non-cellulosic sugars	26.8 ± 4.9	22.0 ± 2.4	9.0 ± 1.5	8.7 ± 1.7	6.6 ± 1.1	7.0 ± 0.48	2.6 ± 0.07	3.8 ± 0.08
C-Glc	16.4 ± 3.3	11.7 ± 2.5	4.5 ± 0.5	6.0 ± 0.1	4.6 ± 0.4	5.3 ± 0.5	1.9 ± 0.1	2.6 ± 0.2

Yields are from one season, with extractions being carried out in triplicate. Sugar composition of non-cellulosic neutral sugars was obtained by TFA hydrolysis. ‘Total non-cellulosic sugars’ are the sum of the neutral sugars plus uronic acid (UA). Cellulosic glucose (C-Glc) content of the cell wall material was obtained by sulphuric acid hydrolysis. Values are means from three cell wall extractions with duplicate assays each ± standard deviation. FW: Fresh weight; Rha: Rhamnose; Fuc: Fucose; Ara: Arabinose; Xyl: Xylose; Man: Mannose; Gal: Galactose; Glc: Glucose; UA: Uronic acid.

The greatest difference between the two cultivars was observed at the fruitlet stage, where the firmer ‘Royal Gala’ fruitlet had about twice the uronic acid content of ‘Scifresh’ but a lower neutral sugar content (Table 1). Although ‘Scifresh’ began with less uronic acid in fruitlet, its relative uronic acid content declined more slowly during growth and maturation than ‘Royal Gala’, up to the point when in cell walls of ripe fruit, ‘Scifresh’ had a higher uronic acid content. In general, during fruit growth and ripening, amounts of all neutral sugars and uronic acid decreased in both cultivars, especially galactose and arabinose.

As there were major changes in the fruit physiology beginning at 70 DAFB in terms of firmness, dry matter and growth rate (Figures 1A, D, E), the percent growth rate (Figure 1E) was compared with the percent change in cell wall material, to determine if the yield of cell wall material was reflected by growth. To make this comparison, net cell wall content was calculated and presented as Additional file 4: Figure S4 showing the difference between percent growth rate and percent loss of cell wall material during growth. This approach showed that the deposition of net cell wall content strongly mirrored growth, in that the rapid growth phase (between 40 and 70 DAFB) was also the phase where net cell wall content was greatest. The two cultivars had a similar pattern of net cell wall content throughout development, with some evidence for greater deposition in ‘Royal Gala’ between 40 and 70 DAFB. Deposition of net cell wall content slowed in both cultivars between 70 and 100 DAFB, and became negligible once the fruit approached maturity.

### Yield, composition and molecular weight distribution of CDTA-soluble pectin differed markedly during growth and ripening

**Figure 8:**
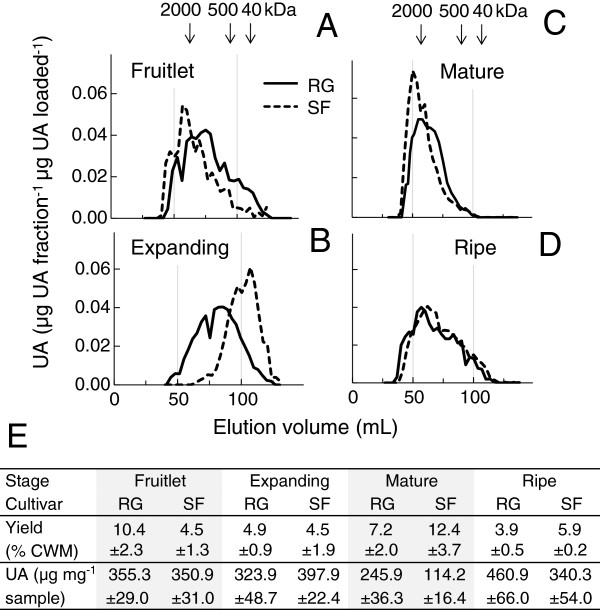
**Size, yield and composition of CDTA-soluble pectin in ‘Royal Gala’ and ‘Scifresh’ fruit during development.** Molecular weight distribution **(A-D)**, yield and uronic acid (UA) content **(E)** of CDTA-soluble pectin in ‘Royal Gala’ (RG) and ‘Scifresh’ (SF). Fruitlet: 40 DAFB; Expanding fruit: 70 DAFB; Mature fruit: 120 DAFB (RG) 140 DAFB (SF); Ripe fruit: 20 weeks at 0.5°C. Yields in **(E)** are means of 3 extractions ± standard deviation; UA values are the mean of 3 CDTA extracts with duplicate assays ± standard deviation.

The molecular weight distribution of the CDTA-soluble pectin differed most between the cultivars during growth and maturation, whereas the composition and yield differed most during the ripening phase (Figure 8). The molecular weight distribution of the CDTA-extracted polyuronates was very similar in fruitlets of both cultivars, but then decreased more in ‘Scifresh’ than ‘Royal Gala’ while fruit expanded (Figure 8A, B). This decrease was accompanied by an increase in yield of CDTA-soluble pectin in ‘Scifresh’ but not in ‘Royal Gala’ (Figure 8E). As the fruit matured, the molecular weight distribution of CDTA-extractable polyuronates increased considerably in ‘Scifresh’ to be similar to ‘Royal Gala’. In ‘Scifresh’, this increase was accompanied by an increase in yield of CDTA-soluble pectin and a decrease in its uronic acid content (Figure 8E). During ripening, the molecular weight distribution broadened and remained comparable between the mature and ripe stage in both cultivars (Figure 8C, D). In both cultivars, yields decreased by a third, whereas the uronic acid content increased per mg polysaccharide (Figure 8E).

### Differences in pectin methylesterase activity early in fruit development may impact the degree of methyl-esterification of cell walls of both cultivars later on in development

**Figure 9:**
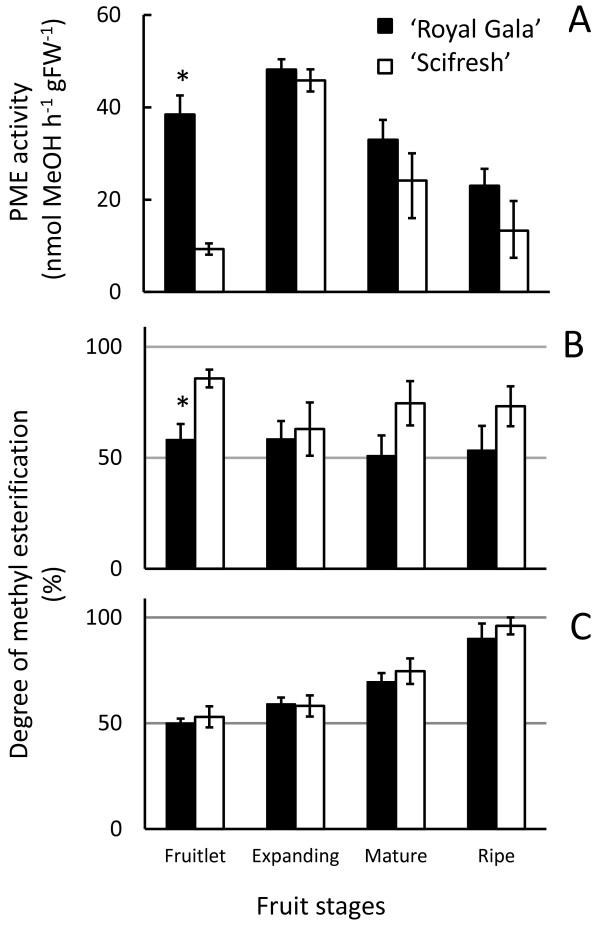
**Pectin methylesterase (PME) activity and degree of methyl esterification of ‘Royal Gala’ and ‘Scifresh’ fruit cell walls during development.** PME activity **(A)**, degree of methyl esterification of cell wall material **(B)** and CDTA-soluble pectin **(C)**. Fruitlet: 40 DAFB; Expanding fruit: 70 DAFB; Mature fruit: 120 DAFB (RG) 140 DAFB (SF); Ripe fruit: 20 weeks at 0.5°C. Values in **(A)** are the means of 3 assays ± standard deviation; values in **(B)** and **(C)** are the means of 6 assays ± standard deviation. Asterisks indicate significantly different values at a level of *P* ≤ 0.05 using a protected Fisher’s least significant difference test.

Significant difference in pectin methylesterase (PME) activity was observed at the fruitlet stage, where ‘Scifresh’ showed about a quarter the activity of ‘Royal Gala’ (Figure 9A). However, while ‘Scifresh’ fruit were expanding, PME activity increased to the same level of activity in ‘Royal Gala’. After the fruitlet stage, PME activity continuously decreased and was similar between the two cultivars.

In general, the degree of methyl-esterification (DE) of cell wall material remained relatively constant during development in both cultivars (Figure 9B). ‘Scifresh’ cell wall material showed generally a higher DE than ‘Royal Gala’ except at the expanding fruit stage, where the DE was similar (Figure 9B). The DE of CDTA-soluble pectin was lowest in fruitlet and increased in both cultivars during development, with DE levels being similar in both cultivars (Figure 9C).

## Discussion

‘Royal Gala’ and ‘Scifresh’ apples share a similar genetic background; ‘Royal Gala’ being a parent of ‘Scifresh’. They have considerable overlap in growth season, and similar physiological characteristics such as growth rate, fruit size, as well as similar starch degradation, soluble solids concentration and equally high ethylene production during ripening. Despite these similarities their cellular microstructure and softening behaviour were quite different. ‘Royal Gala’ was firmer at the fruitlet stage than ‘Scifresh. However, ‘Royal Gala’ lost firmness faster during growth, maturation and ripening, resulting in softer fruit than ‘Scifresh’, which lost little firmness after fruit were mature. These different softening rates were temporally independent of ethylene production, as both apples were high ethylene producers, when compared to other apples [1]. ‘Scifresh’ demonstrated that it is possible for an apple cultivar to have high ethylene production, and yet have a slow softening rate over a long period of time. Our work implies that softening is not solely controlled by ethylene production, but differences in the ethylene signalling pathways, in ethylene sensitivity or in structural features of the cell wall established before the onset of ethylene synthesis may also contribute roles.

Generally, apple fruitlets have small cells with thick, less structured and amorphous cell walls allowing for very rapid expansion [2,25]. In our study, both cultivars had cells about equal in size at the fruitlet stage; however the yield of cell wall material in ‘Scifresh’ was much lower. Hence, cell walls in (softer) ‘Scifresh’ fruitlet must be thinner than cell walls in (firmer) ‘Royal Gala’ fruitlet at this stage. The fruitlet cell walls already showed a different composition, where the firmer ‘Royal Gala’ fruitlet had a higher uronic acid but a lower neutral sugar (side chain) content, accompanied by a higher amount of cellulosic glucose and a lower degree of esterification of pectin than cell walls of ‘Scifresh’ fruitlets. During the cell expansion phase between 40 and 70 DAFB, the yield of cell wall material in both cultivars decreased two to three fold, whereas the fruit size (by mass) increased by 25–30 times. This suggested a “net” synthesis of cell walls during fruit growth, rather than just a gradual thinning of the cell wall as the cells expanded. Although ‘Scifresh’ accumulated more dry weight than ‘Royal Gala’, it had a slightly lower cell wall synthesis rate but seemed to more efficiently sustain stronger cell walls, thereby maintaining the structural firmness of the tissue, as measured by the lag phase in softening during this phase of rapid growth.

In mature fruit, it was evident that ‘Scifresh’ cortical cells had expanded more and were larger in mature fruit compared to ‘Royal Gala’. As the firmness of mature fruit of both cultivars was very similar, it appeared that cell growth and development of larger cells did not necessarily impair cell wall strength and hence tissue firmness. This may also suggest a difference in wall extension dynamics between ‘Royal Gala’ and ‘Scifresh’, which has been shown to be influenced by α-expansins acting on xyloglucans thereby inducing wall loosening [30], as well as extensins, of which a higher level of expression has been linked to a tissue’s ability to withstand higher tensile stresses [31].

‘Scifresh’ not only had the capacity to allocate resources early in fruit development to strengthen the tissue, but also to allow the deposition to occur in a manner that permits more rapid cell expansion. This rapid cell expansion phase has also been previously identified as being a high energy requirement phase where numerous genes are differentially regulated for sugar metabolism and accumulation [32]; and an increase in carbohydrate availability by reduction of fruit load enhanced cell production during early fruit growth [33]. It could be possible that in expanding fruit of ‘Scifresh’ (showing higher dry matter accumulation than ‘Royal Gala’ at this stage), carbon sources in the form of sucrose, fructose or sorbitol are preferentially optimized for structural wall building instead of other cellular processes. Additionally, this could also be indicative of a difference in turnover between metabolized and newly-synthesized sugars in expanding fruit of ‘Royal Gala’ and ‘Scifresh’. Gene expression levels of key enzymes such as expansins, other cell wall-related genes, as well as starch metabolism genes which are differentially regulated across different cultivars of apples [34], warrant more investigations in future studies.

Extraction of cell wall material [35] showed that ‘Scifresh’ fruitlet already had a higher proportion of more tightly bound pectin and hemicelluloses than ‘Royal Gala’, possibly making stronger cellulose-pectin and/or cellulose- hemicellulose networks in ‘Scifresh’ compared to ‘Royal Gala’, cell wall features that continued to exist up to the ripe stage. The observation that ‘Scifresh’ apples can accommodate cell expansion while not compromising structural hardness was in agreement with [36] and [37] who reported that apple cultivars with larger cells have better keeping qualities and ripen slower, presumably due to a lower respiratory rate compared to higher respiration in smaller cells.

Apples with more angular cells have been reported to be firmer [27]. Hexagonal-shaped cells with a higher number of facets have larger areas for adherence compared to spherical-shaped cells [38]. Cells in mature fruit of ‘Scifresh’ were larger and more angular than ‘Royal Gala’, and had a higher cortical density indicating less airspace and therefore greater cell-to-cell contact area. This may also be indicative of a higher turgor pressure, which contributes to higher firmness in apple fruit [5]. Turgor pressure provides non-woody plant tissues with mechanical rigidity and is the driving force for growth, but at the same time it generates large forces tending to separate cells. Therefore, cell adhesion depends on the strength of reinforcing zones located precisely at the points of maximum stress, the cell junctions where adjacent cells meet [39]. At these cell junctions, ‘Scifresh’ was filled with highly esterified pectin as shown by immunolabelling with LM20 from the fruitlet stage throughout development. In ‘Royal Gala’ fruitlet, this labelling was far less intense than in ‘Scifresh’, and perhaps it is the lower presence of pectin in these tricellular junctions that explains the cell size difference observed between the two cultivars.

Strong cell adhesion, less airspace and higher cell density was maintained in ‘Scifresh’ from fruitlet to ripe fruit, whereas fracture surfaces of ripe ‘Royal Gala’ tissue showed less cell rupture and more separation between cells compared to ‘Scifresh’. Cell rupture is a major factor in perception of juiciness, and for this to occur, cell-to-cell adhesion must be strong. Less cell-to-cell contact and more intercellular air spaces have been related to a soft, drier perception of ripe apples [9], which was evident in ripe ‘Royal Gala’ but not in ‘Scifresh’. Moreover, tensile strength or the force to pull tissue apart (which had been the same up to the mature stage) had decreased considerably during ripening of ‘Royal Gala’, but not in ‘Scifresh’. Tensile strength depends on the strength of cell adhesion, but also on the overall strength of the cell wall, i.e. its resistance to fracture or its elasticity [40]. Perhaps one of the driving forces to maintain strong cell adhesion in ‘Scifresh’ even in the ripe stage was the presence of calcium pectate in the corners of tricellular junctions, which was observed by immunolabelling with 2F4 at the mature stage in both fruit, but at the ripe stage was undetectable in ‘Royal Gala’.

The CDTA-soluble fraction of the cell wall contains pectin that is considered an important linking component between adjacent cells, with composition, degree of esterification, and polymer length determining the strength of that ‘glue’. The degree of methyl-esterification (DE) of the CDTA-soluble pectin was very similar between the two cultivars, and slowly increased over development. The increase in DE in the CDTA-soluble fractions did however not necessarily imply biosynthesis as suggested by [41], but rather more highly esterified pectin becomes increasingly extractable with ripening. According to [42], HG with DE higher than 60% (notably present in CDTA-soluble pectin in both cultivars at the mature and ripe stages) can form gels via hydrophobic interactions and hydrogen bonds between methyl-esterified blocks of homogalacturonan. The longer these blocks are, the stronger the gel formed. Although the DE of CDTA-soluble pectin in both cultivars was similar throughout development, and the molecular weight distribution (with the exception of the expanding fruit stage) remained large, yields were different between the cultivars and correlated with fruit firmness throughout growth and ripening, as higher yields were generally found in firmer fruit. Interestingly, in expanding fruit the molecular weight distribution of the CDTA-soluble pectin decreased in both cultivars, but more extensively in ‘Scifresh’ than in ‘Royal Gala’. One could speculate that this decrease in molecular weight distribution may contribute to cell expansion, as the shorter length of these pectin molecules may result in reduced cohesive strength. As pectin molecules were considerably shorter in ‘Scifresh’, ‘Scifresh’ cells may be able to expand more, giving rise to larger cell size compared to ‘Royal Gala’.

In cell wall material, the DE was overall higher in ‘Scifresh’ than in ‘Royal Gala’ throughout development, which coincided with an overall lower PME activity in this cultivar. In agreement with our study, PME in apple has been found to increase during growth and decrease during ripening-related softening [24]. The action of PME affects apoplastic pH and alters the activity of cell wall hydrolases [43] as well as modifies the rigidity of the pectin network [44]. The pattern of methyl ester group distribution within the wall has also been reported to be important in determining pectin properties [42,45]. LM20-labelling of highly-esterified HG regions was more widespread in ‘Scifresh’ cell walls throughout the middle lamella and junction zones, compared to ‘Royal Gala’ where labelling was more restricted to corners of tricellular junctions. This localisation of methyl ester groups in ‘Royal Gala’ could indicate block-like microdomains, which have been suggested to cause a restriction in the movement of enzymes such as PG, which has a preference for lowly-esterified HG [46]. Interestingly, immunodetectable ripening-related PG1 protein was only found in the faster softening ‘Royal Gala’ fruit, but not in ‘Scifresh’, thereby substantiating that PG expression is likely cultivar-dependent and plays a major role in determining apple fruit texture [47,48]. The higher PME activity in ‘Royal Gala’ fruitlet could have resulted in the generation of more non-esterified HG, presenting more substrate for the action of PG, and thus setting the basis for pectin depolymerisation and faster softening in ‘Royal Gala’.

**Figure 10:**
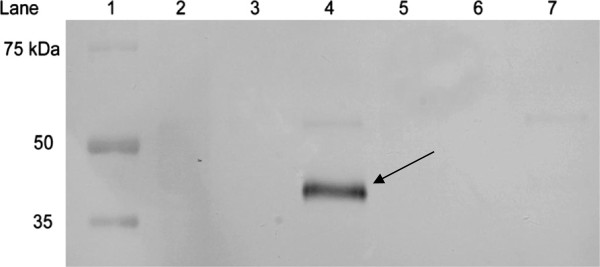
**Western blot stained with a rabbit polyclonal antibody for polygalacturonase.** Lane 1, Precision Plus Dual Protein Standard; lanes 2, 3, 4, ‘Royal Gala’ in the order of fruitlet, mature and ripe fruit; lanes 5, 6, 7, ‘Scifresh’ in the order of fruitlet, mature and ripe fruit. An arrow indicates the presence of PG protein at 45 kDa only detected in ripe ‘Royal Gala’ fruit.

The lack of ripening-related PG1 protein in ripe ‘Scifresh’ (Figure 10) led to some compelling parallels between ‘Scifresh’ and transgenic PG1 antisense ‘Royal Gala’ apples [21]. Like ‘Scifresh’, the antisense apples had increased tensile strength and hence stronger cell adhesion, as well as increased cell rupture compared to wild-type ‘Royal Gala’ at the ripe stage. However, there was no evidence of reduced airspace or higher cell density in the antisense apples, indicating that these features are not related to PG1. The softening rate also decreased in the antisense ‘Royal Gala’ apples compared with the wild-type ‘Royal Gala’ apples; however, they still softened faster than ‘Scifresh’ confirming that there are factors additional to PG involved in apple softening. The differences in cell adhesion between ‘Scifresh’ and PG1 antisense ‘Royal Gala’ on one side and wild-type ‘Royal Gala’ on the other could be related to differences in pectin content. ‘Scifresh’ [35] and antisense ‘Royal Gala’ [21] had higher yields of CDTA-soluble pectin and less water-soluble pectin than wild-type ‘Royal Gala’, implying that PG has some influence on pectin solubilisation.

## Conclusions

We have identified an apple cultivar (‘Scifresh’) where the fruit softens slowly despite having high ethylene production, providing a system for investigating the cell wall and tissue microstructure without the confounding influence of ethylene. Comparisons between ‘Scifresh’ and the more rapidly softening ‘Royal Gala’ identified differences in tissue microstructure and cell wall properties that could influence the rate of softening during ripening. These structural properties were established as early as 40 to 70 days after full bloom and well before the maturation and ripening process are induced. ‘Scifresh’ fruit have larger cells, strong cell-to-cell adhesion and less airspace compared to ‘Royal Gala’. The strong cell-to-cell adhesion appeared to be due to increased deposition of pectin in cell corners, with pectin interactions so strong that very little airspace was able to develop during cell expansion and maturation. The lack of immuno-detectable PG in ‘Scifresh’ most likely strengthened this phenotype. It is probably because of these strong pectin interactions at cell junctions that cells fracture through the cell rather than between cells as in ‘Royal Gala’.

## Methods

### Fruit material and physiological assessment

Apple fruit (*Malus* x *domestica* Borkh.) ‘Scifresh’ and ‘Royal Gala’ were sampled from the Plant & Food Research orchard, Havelock North, New Zealand. At each stage, 20 fruit per cultivar were collected by taking five apples in similar positions from four trees (growing side by side and exposed to the same management regime) throughout this study. Fruit stages were categorised based on number of days after full bloom (DAFB), size, skin colour, starch and soluble solids content. Fruit sampled at 40 DAFB, where fruit were at the end of the cell division phase and at the beginning of cell expansion [2], were termed ‘fruitlet’. Two intermediate time points were sampled during the cell expansion phase, 70 and 100 DAFB, and termed ‘expanding fruit’. Mature ‘Royal Gala’ fruit were sampled at 120 DAFB according to degree of starch clearance, and ‘Scifresh’ at 140 DAFB, as they had a longer developmental period before reaching the same physiological age as ‘Royal Gala’, termed ‘mature’ fruit. These fruit were then ripened at 0.5°C under ambient atmospheric pressure and humidity for 20 weeks and termed ‘ripe fruit’.

Fruit firmness was assessed using puncture [49] and tensile tests [21], with the latter only assessed from 100 DAFB onwards because of fruit size constraints. The puncture test was performed using two cylindrical probes of 5 mm (fruitlet to mature fruit) or 11 mm diameter (mature to ripe fruit), using a TA.XT Plus Texture Analyser (Stable Microsystems, United Kingdom). Internal ethylene concentration [49] and dry matter concentration [50] were determined from three bulked replicates samples from a total pool of 20 fruit. Cortical tissue density was determined in mature fruit by measuring the volume displacement for 1 cm^3^ blocks of excised tissue [4].

After these assessments, fruit tissue from cortex was then diced, immediately frozen in liquid nitrogen and stored at -80°C for cell wall analyses and enzyme activity assays. Tissue preparations for microscopy are described below.

### Conventional scanning electron microscopy (SEM)

Apple sections were cut longitudinally from skin to cortex tissue, not including core tissue, and fixed in 2% paraformaldehyde and 0.1% glutaraldehyde in 0.1 M phosphate buffer (pH 7.2). Segments were washed in phosphate buffer, and dehydrated in an ethanol series from 10% to 100% anhydrous ethanol (in 10% increments). The segments were dried in a Bal-Tec CPD030 critical point dryer (Balzers, Liechtenstein) using liquid CO_2_ as the transitional fluid. The dried material was mounted on an anodized aluminium stub (ProSciTech, Australia) with carbon adhesive tabs (ProSciTech), with edges painted with conductive silver liquid (ProSciTech), and left to air dry for 1 h. The material was sputter-coated with gold in a SEM coating unit E5100 (Polaron equipment Ltd, England) and stored over silica beads (Scharlau, Spain) in an air-tight container until imaged. Scanning electron microscopy was carried out using a Quanta 250 SEM (FEI, Hillsboro, USA), with accelerating voltage of 15 kV.

### Cryo-scanning electron microscopy

Cryo-SEM was performed using a Polaron PP2000 Cryo Transfer system (QuorumTechnologies, United Kingdom) attached to a FEI Quanta 250 Scanning Electron Microscope [51]. ‘Royal Gala’ and ‘Scifresh’ apples were matched for size, and samples of cortex tissue were cut and mounted in sample holders containing a mixture of colloidal graphite and OCT™ compound (Sakursa Finetek, Netherlands). Tissue was immediately frozen in liquid nitrogen slush, transferred to the preparation chamber of the PP2000 system where the tissue was fractured to expose the surface for viewing. Ice was sublimed away to partially etch the surface at a temperature of -90°C for 15 minutes, sputter-coated with gold/palladium, transferred to the cryo-stage in the SEM (-150°C) and observed at an accelerating voltage of 10 kV.

### Immunolabelling

Fixed and ethanol-dehydrated sections prepared as described above were infiltrated with LR White resin (London Resin Company Ltd, United Kingdom) and placed in gelatine capsules (ProSciTech) containing LR White resin [52]. After hardening (55°C, 48 h), the capsule was removed, embedded tissue sectioned using a diamond knife and Leica UCT ultramicrotome (Leica, Germany), and sections (200 nm) air dried onto Superfrost® poly-L-lysine slides (25× 75× 1 mm, Biolab, USA). Antibodies used for labelling were LM19, LM20 [53] and 2F4, all supplied by PlantProbes (United Kingdom).

For labelling with LM19 and LM20, sections were wetted with phosphate-buffered saline with 0.1% Tween 80 (PBS-T) for 10 min, then incubated with 0.1% bovine serum albumin c (BSA-c; Aurion, Netherlands) in PBS-T to block non-specific labelling (15 min), followed by incubation with primary antibody (dilution 1:20 v/v in 0.1% BSA-c in PBS-T) overnight at 4°C in a moist chamber. Slides were then washed in PBS-T.

For 2F4, a chemical de-esterification step prior to immunolabelling was necessary to unmask epitopes. Briefly, the sections were incubated in 0.05 M NaOH (pH 12.4) for 30 min at room temperature, followed by BSA-c as described above, for blocking of non-specific binding. Although this treatment may introduce artefacts or in some way alter the original abundance of epitope-antigen binding sites, because the same procedures were carried out on both apple cultivars, this makes them comparable. The sections were then incubated in TCaS buffer (20 mM Tris-HCl pH 8.2, 1 mM CaCl2 , 150 mM NaCl) containing 5% (w/v) low fat milk powder for 1 h to block non-specific labelling, followed by incubation with primary antibody 2F4 [dilution 1:20 (v/v)] in TCaS buffer with 0.05% (v/v) Tween 20 and 1% (w/v) low fat milk powder overnight at 4°C in a moist chamber, and the slides washed in TCaS Buffer [54].

Labelling with the secondary antibody was the same for all samples. The slides were incubated for 2 h in the dark at room temperature with goat anti-rat IgG AlexaFluor 488 (Molecular Probes, Oregon, USA) diluted 1: 600 (v/v) in PBS.

Sections were further incubated in 1 mL of 0.01% calcofluor (Fluorescent Brightener 28, Sigma) for 6 min at room temperature [55] to distinguish the boundaries of cell walls where there may be absence of antibody labelling. Sections were washed in ultrapure water, allowed to dry at room temperature and coverslip-mounted onto the slide using anti-fade agent Citifluor (Citifluor, United Kingdom) [52].

Negative controls were carried out using only the secondary antibody omitting the primary antibody (200 μL of goat anti-rat IgG AlexaFluor 488 diluted 1: 600 in PBS), where sections were incubated for 2 h in the dark before imaging, results were negative (images not shown).

Sections were viewed using an Olympus Vanox AHT3 compound microscope (Olympus Optical, Tokyo, Japan) with a blue-interference filter set for antibody labelling and UV filterset for calcofluor staining and imaged with a CoolSnap colour digital camera system (Photometrics, USA). Images were further processed using Adobe Photoshop Version 6.0 on Windows XP. Two sets of images are shown; higher magnification dual-labelling with calcofluor (blue) and antibody labelling contrast-enhanced by changing the hue angle to a reddish-pink colour (Figures 5, 6), and antibody immunofluorescence labelling in green (Figure 7 and Additional file 1: Figure S1, Additional file 2: Figure S2, Additional file 3: Figure S3).

### Preparation of cell wall material and CDTA-soluble pectin

Cell wall isolation and extractions were performed using a composite sample of cortical tissue from 20 apples that was divided into three sub-samples of fruit to form three extraction replicates. Cell wall preparations were carried out as described in [56] to give the water-soluble fraction and cell wall material, with the exception that after dimethyl sulphoxide extraction, residual starch in fruitlet was removed by digestion with α-amylase (40 U/mL) (Megazyme, Ireland) and pullulanase (20 U/mL) (Megazyme) by incubation at 37°C for 1 h. CDTA-soluble pectin was extracted from cell wall material (2.5 g) as described in [56].

### Size exclusion chromatography

CDTA- soluble pectin (2.5 mg) was dissolved in 0.5 mL water and eluted through a column (2.5× 100 cm) of Sepharose CL–2B (GE Healthcare, USA), in 0.05 M ammonium acetate buffer (pH 5.0) with an average flow rate of 5 mL h^-1^. Fractions (20 min) were collected and assayed for uronic acid [57]. The column was calibrated with dextrans (GE Healthcare) Blue Dextran (2 MDa), T500 (500 kDa) and T40 (40 kDa).

### Pectin methylesterase (PME) extraction and activity assay

Ground frozen apple tissue (0.25 g) was extracted in 0.5 mL of 0.2 M MES, 7.5 mM potassium tetrathionate, 10 mM dithiothreitol, 1.7 M NaCl (pH 6.0) with 25 mg of polyvinylpolypyrrolidone. The mixture was vortexed, centrifuged and supernatant recovered. The pellet was re-suspended in 0.25 mL of the extraction buffer and incubated on ice for 20 min, and supernatant recovered as described above. The combined supernatant was the PME extract. PME activity was assayed [58], whereby the amount of methanol released is quantified by reacting with alcohol oxidase and N-methylbenzothiazolinone-2-hydrazone. The reaction mixture contained PME extract (10 μL), 100 mM Tris-HCl pH 7.5 (20 μL), 0.5 U/mL alcohol oxidase (5 μL; *Pischia pastoris*, Sigma-Aldrich), 3 mg · mL^-1^ N-methylbenzothiazolinone-2-hydrazone (8 μL; Merck, USA), H_2_O (7 μL) and 0.5 mg · mL^-1^ esterified citrus pectin (DE >85%; Sigma-Aldrich) in 50 mM NaCl pH 7.0 (10 μL). After incubation at 30°C for 20 min, the reaction was terminated by addition of 40 μL of 5 mg · mL^-1^ ferrous ammonium sulphate (BDH, United Kingdom) in sulphamic acid (Medica Pacifica, NZ) and absorbance read at 620 nm. PME activity was expressed as moles methanol released per hour per gram fresh weight, based on a standard curve using methanol. Activity assays were carried out in triplicate.

### Polygalacturonase (PG) extraction and Western blotting

Protein was extracted from ground frozen apple tissue (0.1 g) in 1 mL of 7 M urea, 2 M thiourea, 40 mM Tris, 75 mM DTT, 4% CHAPS and concentrated by cold acetone precipitation. Proteins were separated by sodium dodecyl sulphate polyacrylamide gel electrophoresis (SDS-PAGE) (Mini-PROTEAN ® TGX™, BIO-RAD, USA), electroblotted onto a polyvinyldifluoride membrane, and blocked as described in [21]. Proteins were immuno-labelled with the antiserum raised to apple PG1 (1: 1000 (v/v), diluted in TBS buffer containing 5% non-fat milk powder). Membranes were incubated with an anti-rabbit alkaline phosphatase conjugated secondary antibody (Sigma-Aldrich) and PG binding visualised using 1-Step™ NBT/BCIP (Thermo Scientific, USA).

### Analytical methods

Uronic acids were measured as described in [57]. The degree of methyl esterification (DE) was determined by a modified method from [59] by gas chromatographic quantification of methanol after saponification of pectin. Polysaccharides were saponified overnight at 4°C in a solution of 50 mM citric acid, 1 M NaCl and 1 M NaOH. The mixture was neutralised with citric acid and 25 mM *n*-propanol added as the internal standard. Samples (1 μL) were analysed by GC-FID on a BP20 column [(15 m × 0.25 μm) Fischer Scientific, UK; oven temperature 80°C, helium flow rate 1.5 psi; detector temperature at 240°C], with measurements carried out in duplicate. DE was calculated as a molar ratio of methanol to uronic acid, based on a standard curve constructed with known amounts of methanol.

### Statistical analysis

Single-factor (for firmness and yield of cell wall material) or two-way (for compositional and enzyme assay results) ANOVA (analysis of variance) analyses were conducted using the Microsoft Excel 2007 for Windows software. Means were compared using the Fisher’s least significant difference (LSD) post-test at *P* ≤ 0.05 using the SPSS (Version 15.0) software (IBM, USA).

## Abbreviations

CDTA: Trans-1,2-diaminocyclohexane-N,N,N',N'-tetraacetic acid; DAFB: Days after full bloom; DE: Degree of methyl esterification; DTT: Dithiothreitol; HG: Homogalacturonan; LM: Leeds monoclonal antibody; PBS-T: Phosphate-buffered saline containing 0.1% Tween80; PG: Polygalacturonase; PME: Pectin methylesterase; SD: Standard deviation; SE: Standard error; UA: Uronic acid.

## Competing interests

The authors declare that they have no competing interests.

## Authors’ contributions

JWJ, LDM, BGS, RS, ICH and JN conceived of the study and designed the experiments, JN carried out all experiments, with assistance from MIH and RP for fruit assessments and enzyme assays, and PWS for immunolabelling. RS and JN carried out data analyses, and JN, RS, JWJ, BGS, ICH, and LDM wrote the paper. All authors read and approved the final manuscript.

## Supplementary Material

Additional file 1: Figure S1 Immunofluorescence labelling of lowly-esterified homogalacturonan with antibody LM19 in ‘Royal Gala’ (A-D) and ‘Scifresh’ (E-H) apple cortex tissue. Fruitlet: 40 DAFB; Expanding fruit: 70 DAFB; Mature fruit: 120 DAFB (RG) 140 DAFB (SF); Ripe fruit: 20 weeks at 0.5°C. Bar in (A) = 50 μm for all micrographs. is: intercellular space. Click here for file

Additional file 2: Figure S2 Immunofluorescence labelling of highly-esterified homogalacturonan with antibody LM20 in ‘Royal Gala’ (A-D) and ‘Scifresh’ (E-H) apple cortex tissue. Fruitlet: 40 DAFB; Expanding fruit: 70 DAFB; Mature fruit: 120 DAFB (RG) 140 DAFB (SF); Ripe fruit: 20 weeks at 0.5°C. Bar in (A) = 50 μm for all micrographs. is: intercellular space. Click here for file

Additional file 3: Figure S3 Immunofluorescence labelling of highly-esterified homogalacturonan with antibody LM20 in ‘Royal Gala’ (A) and ‘Scifresh’ (B, C) fruitlet cortex tissue in high magnification. Bars = 10 μm for all micrographs. Panel A shows ‘Royal Gala’ fruitlet section with distinct LM20-labelling pattern concentrated at the corners (cr) of tricellular junctions and very intense staining in the middle lamella (ml) regions particularly the lining of the intercellular air space (lis). Panels B and C are ‘Scifresh’ fruitlet sections showing a different labelling pattern to ‘Royal Gala’. Panel B shows a tricellular junction with intense LM20-labelling completely filling this area. In all sections viewed, 70-80% of tricellular junctions in ‘Scifresh’ fruitlet were completely stained, while only 30-40% in ‘Royal Gala’ fruitlet displayed this pattern. Panel C shows a ‘Scifresh’ junction zone located between 5 cells labelled with LM20, however the lining of the intercellular air space (lis) was absent of labelling, which was opposite to the pattern observed in ‘Royal Gala’ fruitlet (A). This emphasizes the different localisation of highly-esterified homogalacturonan in the cell walls of the two apple cultivars. Click here for file

Additional file 4: Figure S4 Percent increment in net cell wall deposition of ‘Royal Gala’ and ‘Scifresh’ per day. Data based on Figure 1E and Table 1, with the percentage increase in fruit weight or increase in cell wall material calculated as the change in mean fruit weight or mean yield of cell wall material relative to the weight or yield of cell wall material, respectively at the start of each period per day. Click here for file
