# Supplementary figures and images for: Cell wall structures leading to cultivar differences in softening rates develop early during apple (Malus x domestica) fruit growth
Source: BMC Plant Biol. 2013 Nov 19;13:183. doi: 10.1186/1471-2229-13-183 (PMC4225529; doi:10.1186/1471-2229-13-183)

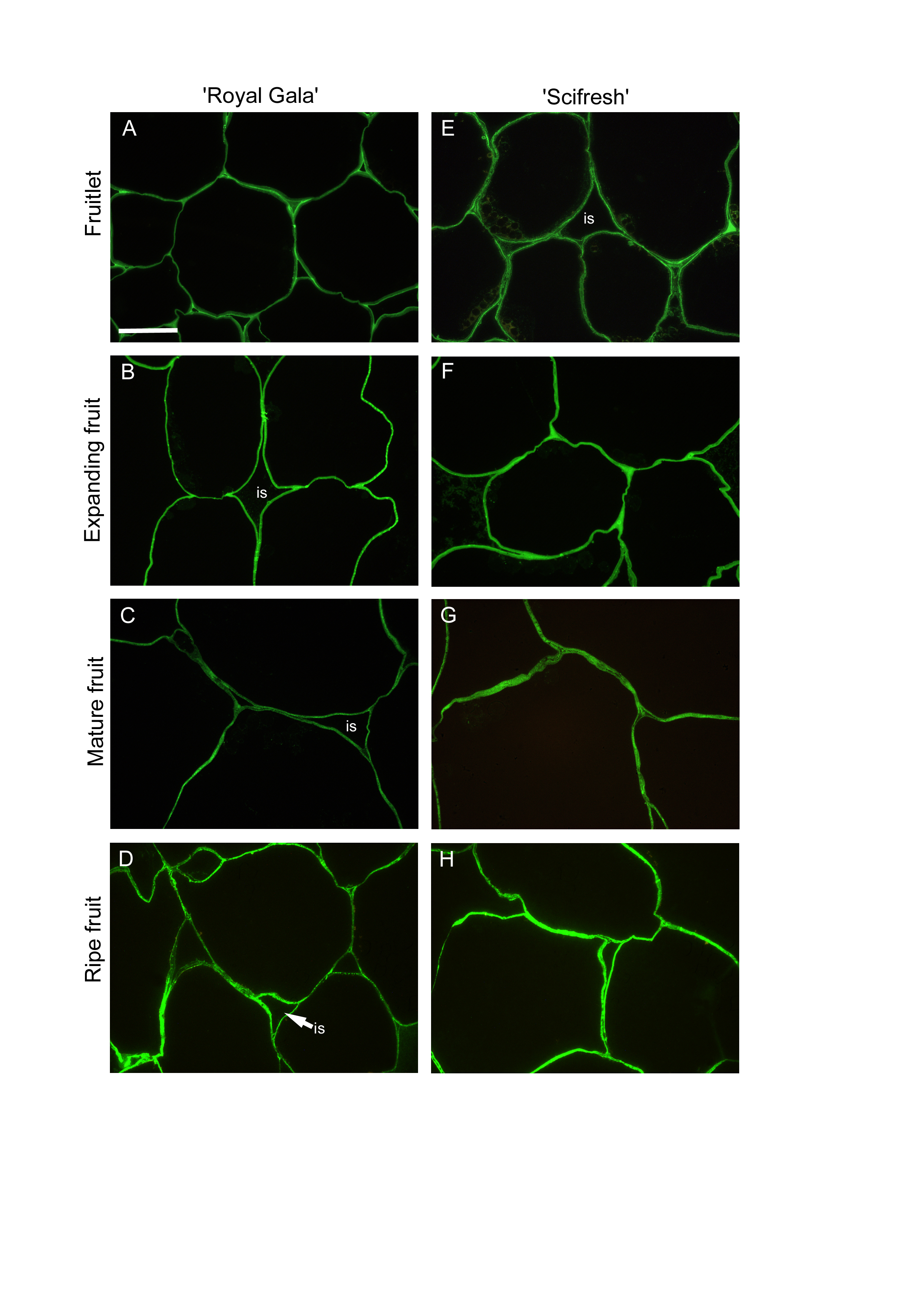

Supplement: Additional file 1: Figure S1 — Immunofluorescence labelling of lowly-esterified homogalacturonan with antibody LM19 in ‘Royal Gala’ (A-D) and ‘Scifresh’ (E-H) apple cortex tissue. Fruitlet: 40 DAFB; Expanding fruit: 70 DAFB; Mature fruit: 120 DAFB (RG) 140 DAFB (SF); Ripe fruit: 20 weeks at 0.5°C. Bar in (A) = 50 μm for all micrographs. is: intercellular space. [file 1471-2229-13-183-S1.jpeg]

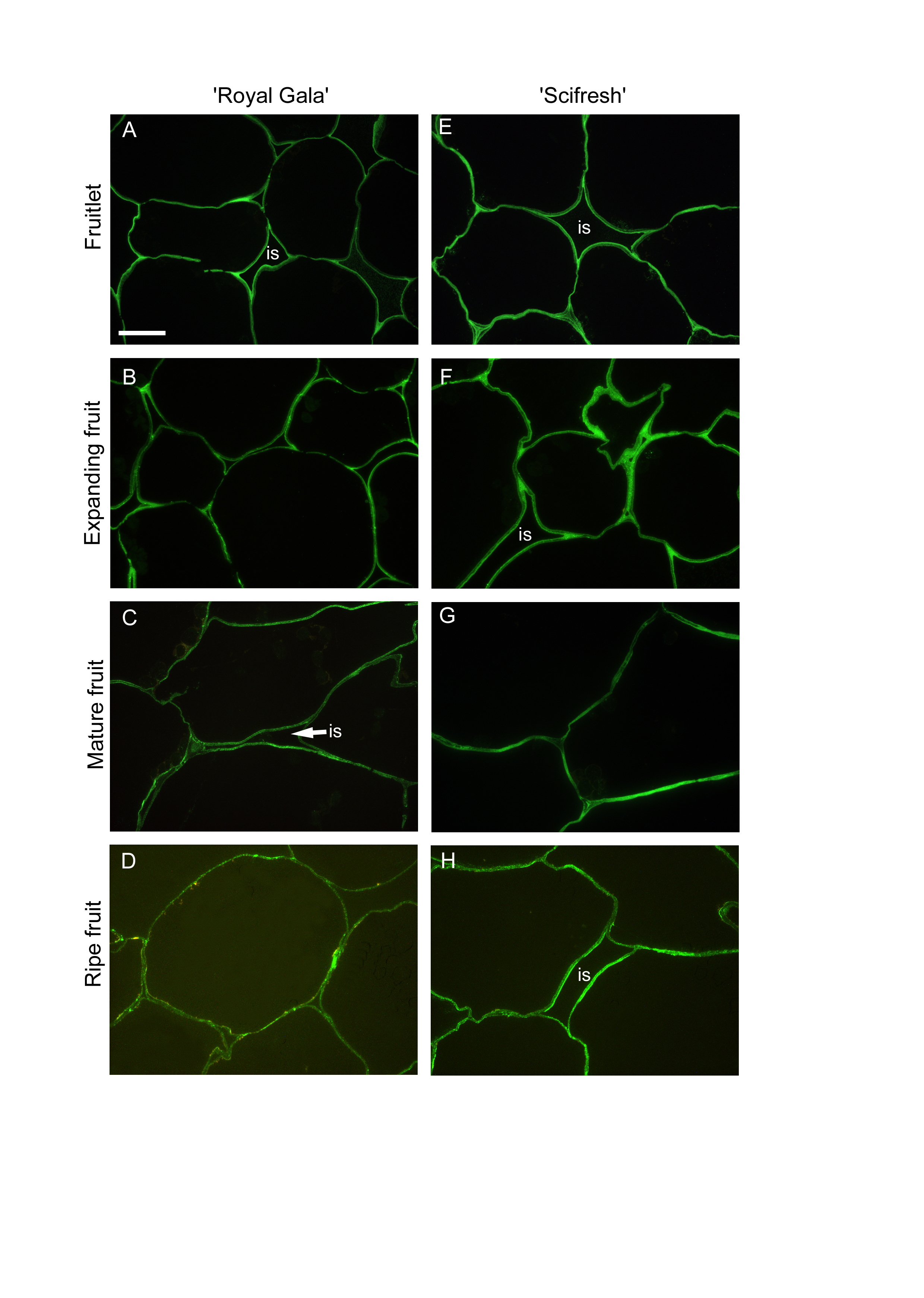

Supplement: Additional file 2: Figure S2 — Immunofluorescence labelling of highly-esterified homogalacturonan with antibody LM20 in ‘Royal Gala’ (A-D) and ‘Scifresh’ (E-H) apple cortex tissue. Fruitlet: 40 DAFB; Expanding fruit: 70 DAFB; Mature fruit: 120 DAFB (RG) 140 DAFB (SF); Ripe fruit: 20 weeks at 0.5°C. Bar in (A) = 50 μm for all micrographs. is: intercellular space. [file 1471-2229-13-183-S2.jpeg]

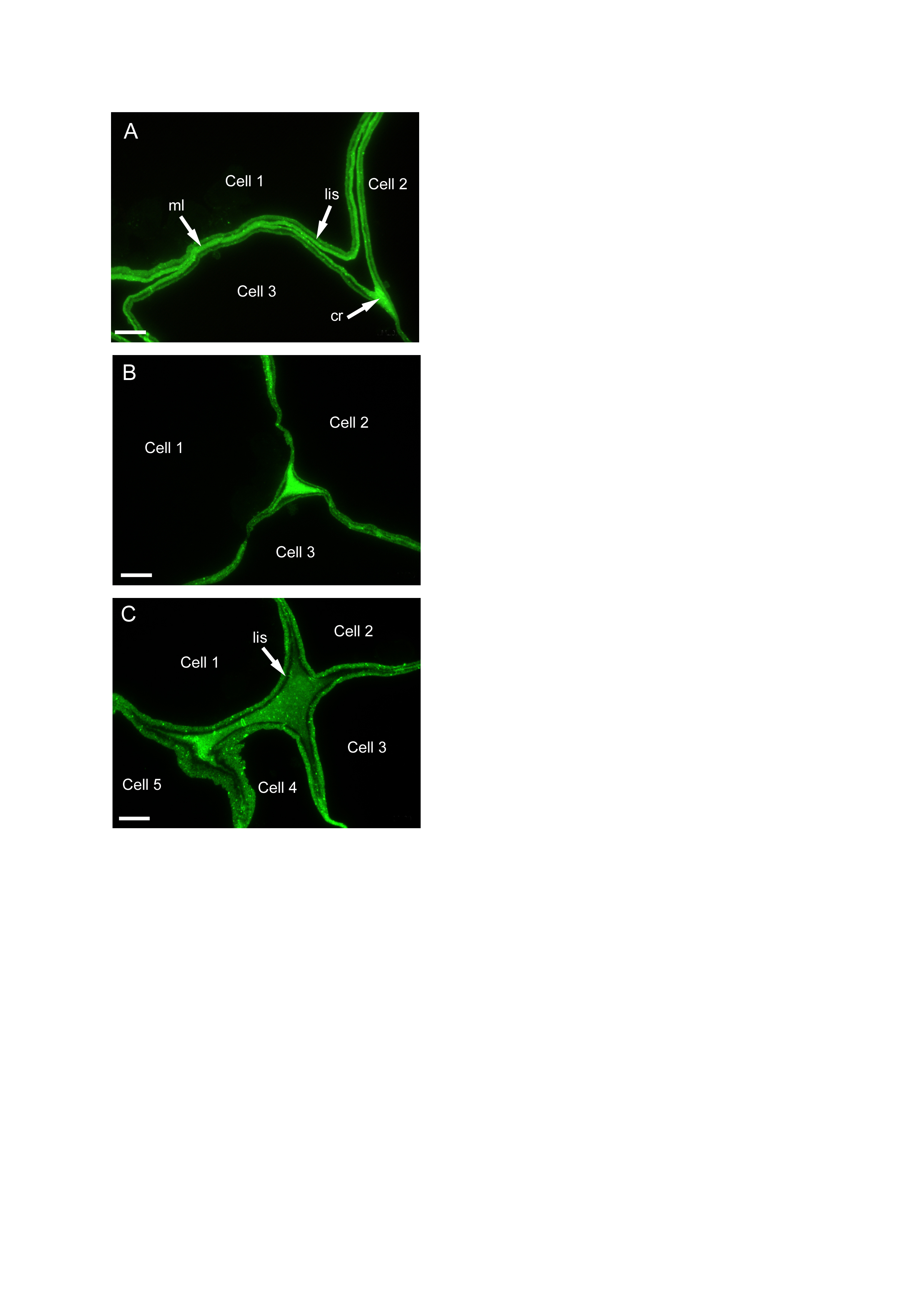

Supplement: Additional file 3: Figure S3 — Immunofluorescence labelling of highly-esterified homogalacturonan with antibody LM20 in ‘Royal Gala’ (A) and ‘Scifresh’ (B, C) fruitlet cortex tissue in high magnification. Bars = 10 μm for all micrographs. Panel A shows ‘Royal Gala’ fruitlet section with distinct LM20-labelling pattern concentrated at the corners (cr) of tricellular junctions and very intense staining in the middle lamella (ml) regions particularly the lining of the intercellular air space (lis). Panels B and C are ‘Scifresh’ fruitlet sections showing a different labelling pattern to ‘Royal Gala’. Panel B shows a tricellular junction with intense LM20-labelling completely filling this area. In all sections viewed, 70-80% of tricellular junctions in ‘Scifresh’ fruitlet were completely stained, while only 30-40% in ‘Royal Gala’ fruitlet displayed this pattern. Panel C shows a ‘Scifresh’ junction zone located between 5 cells labelled with LM20, however the lining of the intercellular air space (lis) was absent of labelling, which was opposite to the pattern observed in ‘Royal Gala’ fruitlet (A). This emphasizes the different localisation of highly-esterified homogalacturonan in the cell walls of the two apple cultivars. [file 1471-2229-13-183-S3.jpeg]

## Slide 1
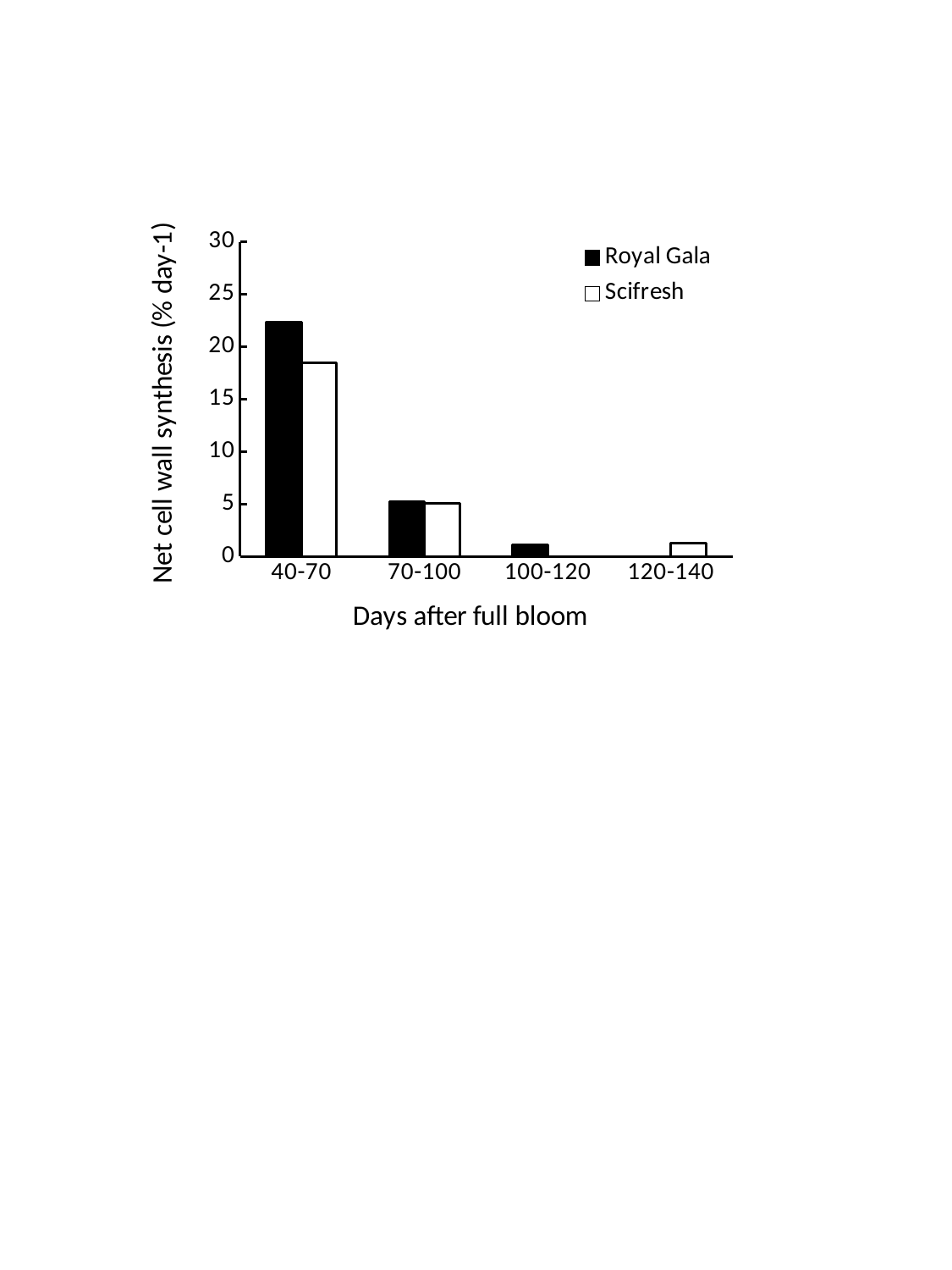

### Chart
| Category | Royal Gala | Scifresh |
|---|---|---|
| 40-70 | 22.316766122642214 | 18.454849258679477 |
| 70-100 | 5.238980979834291 | 5.071623277559052 |
| 100-120 | 1.10305107863392 | -0.09243746013424943 |
| 120-140 | None | 1.291415626821162 |

Supplement: Additional file 4: Figure S4 — Percent increment in net cell wall deposition of ‘Royal Gala’ and ‘Scifresh’ per day. Data based on Figure 1E and Table 1, with the percentage increase in fruit weight or increase in cell wall material calculated as the change in mean fruit weight or mean yield of cell wall material relative to the weight or yield of cell wall material, respectively at the start of each period per day. [file 1471-2229-13-183-S4.pptx]
